# NitroDIGE analysis reveals inhibition of protein *S*-nitrosylation by epigallocatechin gallates in lipopolysaccharide-stimulated microglial cells

**DOI:** 10.1186/1742-2094-11-17

**Published:** 2014-01-28

**Authors:** Zhe Qu, Fanjun Meng, Hui Zhou, Jilong Li, Quanhui Wang, Fan Wei, Jianlin Cheng, C Michael Greenlief, Dennis B Lubahn, Grace Y Sun, Siqi Liu, Zezong Gu

**Affiliations:** 1 Department of Pathology & Anatomical Sciences, University of Missouri School of Medicine, Columbia, MO 65212, USA; 2 Center for Translational Neuroscience, University of Missouri School of Medicine, Columbia, MO 65212, USA; 3 Department of Biochemistry, University of Missouri School of Medicine, Columbia, MO 65211, USA; 4 Department of Computer Science, Informatics Institute, University of Missouri, Columbia, MO 65211, USA; 5 Department of Chemistry, University of Missouri, Columbia, MO 65211, USA; 6 Beijing Institute of Genomics, Chinese Academy of Sciences, Beijing 100101, China

**Keywords:** Epigallocatechin-3-gallate, Lipopolysaccharides, Microglia, Neuroinflammation, Nitric oxide, *S*-Nitrosylation

## Abstract

**Background:**

Nitric oxide (NO) is a signaling molecule regulating numerous cellular functions in development and disease. In the brain, neuronal injury or neuroinflammation can lead to microglial activation, which induces NO production. NO can react with critical cysteine thiols of target proteins forming *S*-nitroso-proteins. This modification, known as *S*-nitrosylation, is an evolutionarily conserved redox-based post-translational modification (PTM) of specific proteins analogous to phosphorylation. In this study, we describe a protocol for analyzing *S*-nitrosylation of proteins using a gel-based proteomic approach and use it to investigate the modes of action of a botanical compound found in green tea, epigallocatechin-3-gallate (EGCG), on protein *S*-nitrosylation after microglial activation.

**Methods/Results:**

To globally and quantitatively analyze NO-induced protein *S*-nitrosylation, the sensitive gel-based proteomic method, termed NitroDIGE, was developed by combining two-dimensional differential in-gel electrophoresis (2-D DIGE) with the modified biotin switch technique (BST) using fluorescence-tagged CyDye™ thiol reactive agents to label *S*-nitrosothiols. The NitroDIGE method showed high specificity and sensitivity in detecting *S*-nitrosylated proteins (SNO-proteins). Using this approach, we identified a subset of SNO-proteins *ex vivo* by exposing immortalized murine BV-2 microglial cells to a physiological NO donor, or *in vivo* by exposing BV-2 cells to endotoxin lipopolysaccharides (LPS) to induce a proinflammatory response. Moreover, EGCG was shown to attenuate *S*-nitrosylation of proteins after LPS-induced activation of microglial cells primarily by modulation of the nuclear factor erythroid 2-related factor 2 (Nrf2)-mediated oxidative stress response.

**Conclusions:**

These results demonstrate that NitroDIGE is an effective proteomic strategy for “top-down” quantitative analysis of protein *S*-nitrosylation in multi-group samples in response to nitrosative stress due to excessive generation of NO in cells. Using this approach, we have revealed the ability of EGCG to down-regulate protein *S*-nitrosylation in LPS-stimulated BV-2 microglial cells, consistent with its known antioxidant effects.

## Background

Nitric oxide (NO) is a signaling molecule that regulates diverse biological processes. As a neuromodulator in the nervous system, NO is involved in brain development, neuronal plasticity, and synaptic neurotransmission. While many intracellular and extracellular molecules may participate in neuronal injury, accumulation of nitrosative stress due to excessive generation of NO appears to be a potential factor contributing to a variety of neurodegenerative diseases, including Parkinson’s disease, Alzheimer’s disease, brain injury, and stroke [1-5]. An important role for NO is its involvement in *S*-nitrosylation, a form of protein modification coupling NO to a reactive cysteine thiol to form *S*-nitrosothiol. *S*-Nitrosylation is a prototypical, redox-based post-translational modification (PTM) akin to phosphorylation. Substantial evidence indicates that protein *S*-nitrosylation controls a number of cellular signaling and/or protein activities by regulating protein misfolding, degradation, mitochondrial fragmentation, and apoptosis [5-9].

Studies investigating protein *S*-nitrosylation are challenging because of the low abundance of *S*-nitrosylated proteins (SNO-proteins) and the lability of *S*-nitrosothiol in the presence of light or metal ions such as Mg^2+^, Ca^2+^, and Cu^2+^. Previously, *in vivo* protein *S*-nitrosylation was detected using the biotin switch technique (BST) [3,10]. This method requires three steps: blocking free cysteine thiols with sulfhydryl-reactive reagents, such as methyl methanethiosulfonate (MMTS), converting *S*-nitrosothiols to thiols with ascorbate, and biotinylating nascent thiols with N-[6-(biotinamido)hexyl]-3’-(2’-pyridyldithio) propionamide (Biotin-HPDP), followed by avidin-agarose pull-down and immunoblotting of the proteins of interest. Despite having furthered the investigation of SNO-proteins [3,6-14], BST is a relatively low-throughput method. Cysteine biotinylation with Biotin-HPDP is rather unstable in reducing conditions, and thus is difficult to use in quantitative analysis. To quantify redox-based *S*-nitrosylation in a context relevant to physiological and pathological conditions, more effective methods are urgently needed.

In this study, we developed a gel-based proteomic approach to screen protein *S*-nitrosylation. This approach, termed NitroDIGE, is a modification of the BST method, combining it with two-dimensional differential in-gel electrophoresis (2-D DIGE) [15]. Fluorescence-tagged CyDye™ thiol reactive agents, Cy3 and Cy5, were used to specifically label *S*-nitrosylated cysteines of proteins. CyDye™ DIGE Cy3 and Cy5 fluorescence dyes have a cysteine thiol-reactive maleimide group, which reacts rapidly with free cysteine thiols to form stable thioether bonds. After CyDye™ labeling, changes in protein *S*-nitrosylation among different samples were quantified on 2-D DIGE gels by measuring the CyDye™ fluorescence intensity.

Microglial cells are associated with innate immune responses in the nervous system. Activation of microglial cells by intrinsic or extrinsic factors results in production of inflammatory mediators, such as tumor necrosis factor-α and interleukin-1, as well as free radicals and NO via activation of inducible NO synthase. Immortalized murine BV-2 microglial cells are known to be activated by lipopolysaccharides (LPS), producing excessive NO and triggering proinflammatory responses including protein *S*-nitrosylation [16-23]. Activation of microglial cells has been implicated in neuroinflammation underlying brain injury and neurodegenerative diseases. Agents that inhibit microglial activation may have broad utility in treating diseases accompanied by neurodegeneration and neuroinflammation [24]. Epigallocatechin-3-gallate (EGCG), a polyphenol from green tea, has been shown to inhibit microglial activation in Parkinson’s disease, Alzheimer’s disease, and amyotrophic lateral sclerosis [25-29]. However, the underlying protective mechanism remains unclear. Here, we applied the NitroDIGE method to investigate SNO-proteins in LPS-stimulated BV-2 cells and to further evaluate the effects of EGCG on protein *S*-nitrosylation under microglial activation.

## Materials and Methods

### Materials

Dulbecco’s modified Eagle’s medium (DMEM) was obtained from Life Technologies-Invitrogen (Carlsbad, CA, USA). Fetal bovine serum (FBS) was purchased from Atlanta Biologicals, Inc. (Lawrenceville, GA, USA). Dithiothreitol (DTT), iodoacetamide, *2*-mercaptoethanol, MMTS, neocuproine, [3-(4,5-dimethylthiazol-2-yl)-2,5-diphenyl-2H-tetrazolium bromide (MTT), LPS from *Escherichia coli* F583 (Rd mutant), ProteoSilver™ Silver Stain Kit, protease inhibitor cocktails, anti-rabbit IgG-peroxidase antibody produced in goat (A0545), and anti-mouse IgG-peroxidase antibody produced in goat (A0168) were purchased from Sigma-Aldrich (St. Louis, MO, USA). CyDye™ DIGE Fluor saturation dyes, IPG buffer (pH 3–10) and Immobiline™ DryStrip gels (24-cm, pH 3–10) were obtained from GE Healthcare (Buckinghamshire, UK). Trypsin (modified, sequencing grade) was obtained from Promega (Madison, WI, USA). The Bicinchoninic Acid Protein Assay Kit, Biotin-HPDP, and NeutrAvidin agarose resin were purchased from ThermoFisher Scientific-Pierce (Rockford, IL, USA). Superoxide dismutase 2 (SOD2) antibody (ab13534) was obtained from Abcam (Cambridge, UK). Peroxiredoxin (PRDX) antibody (sc-33574) and ubiquitin carboxyl-terminal hydrolase 14 (USP14) antibody (sc-100630) were obtained from Santa Cruz Biotechnology (Santa Cruz, CA, USA).

### Cell culture

BV-2 cells were cultured in DMEM containing 5% heat-inactivated FBS and maintained at 37°C in a humid atmosphere containing 95% air and 5% CO_2_ as previously described [19].

### Protein *S*-nitrosylation

A 100 mM stock solution of a physiological NO donor *S*-nitrosocysteine (SNOC) was freshly prepared. For *in vitro S*-nitrosylation, BV-2 cell lysates were treated with various amounts of SNOC (10, 20, 40, 80, or 200 μM) for 30 minutes at room temperature. For *ex vivo S*-nitrosylation, BV-2 cells were exposed to 20 μM SNOC in FBS-free DMEM and incubated at 37°C for 30 minutes. For *in vivo S*-nitrosylation, BV-2 cells were starved for 4 hours after replenishment with FBS-free DMEM (without phenol red). The cells were then exposed to 100 ng/mL LPS for 20 hours to induce NO production. To examine the action of the green tea active component, 10 μM EGCG was added to the medium 1 hour prior to LPS exposure.

### MTT cell viability assay

BV-2 cells were cultured in 24-well plates and treated with LPS, and/or EGCG at different doses or different time courses. The medium was then removed and replaced with 500 μL of DMEM containing 0.5 mg/mL MTT. After incubation at 37°C for 4 hours, formazan crystals were precipitated and re-dissolved in 500 μL DMSO. The absorbance at 540 nm was read using a Synergy-4 micro-plate reader (BioTek Instruments, Inc., Winooski, VT, USA).

### Griess reaction for NO measurement

NO was measured by detecting its nitrite byproduct; 1% sulfanilamide in 5% phosphoric acid and 0.1% N-1-napthylethylenediamine dihydrochloride in water were prepared as stock solutions and mixed 1:1 (v:v) as a working solution just before use. Following LPS stimulation, conditioned medium was collected and mixed with an equal volume of the working solution. After a 10-minute incubation at room temperature, absorbance at 543 nm was measured using the BioTek Synergy 4 micro-plate reader. A sodium nitrite dilution series (0, 5, 10, 25, 50, and 100 μM) was used to generate a nitrite standard reference curve to calculate NO concentration [30].

### BST protocol

For the BST protocol [3], free cysteines in samples were blocked with MMTS. Samples were then acetone-precipitated and dissolved in Hepes/EDTA/Neocuproine (HEN) buffer containing 1% SDS, 5 mM sodium ascorbate, and 0.2 mM Biotin-HPDP. After incubation at room temperature for 1 hour in the dark, excess Biotin-HPDP was removed by acetone precipitation. Biotinylated proteins were enriched by pull-down with NeutrAvidin agarose resin, and eluted with SDS-PAGE sample buffer containing 100 mM 2-mercaptoethanol. Eluates were subjected to immunoblotting for detection of each protein of interest.

### NitroDIGE detection of SNO-proteins

After various treatments, cells were lysed in HEN buffer, pH 7.4, containing 1% Triton X-100, 0.1% SDS, and 1% of a protease inhibitor cocktail. Protein concentration was determined using a bicinchoninic acid protein assay kit and adjusted to 1 mg/mL. Free thiols were blocked with 4X volume of 20 mM MMTS in HEN buffer containing 2.5% SDS at 50°C for 30 minutes. Excess MMTS was removed by precipitation with a 2X volume of cold acetone for 30 minutes. Protein pellets were washed, dissolved in HEN buffer containing 1% SDS and 5 mM sodium ascorbate, and incubated at room temperature for 1 hour. After precipitation, proteins were dissolved in labeling buffer (30 mM Tris-Cl, pH 7.4, 8 M urea, 4% CHAPS) at 2.5 mg/mL. Then, 10 μM CyDye™ DIGE Fluor reagent (either Cy3 or Cy5) was added to each sample and incubated at room temperature for 1 hour to label NO-released thiols. Each group consisted of at least three biological replicates; each replicate was labeled with Cy5, and a mixture containing an equal amount of all samples was labeled with Cy3 to serve as the internal standard. After quenching with 50 mM DTT, labeled samples (internal standard versus each replicate) were mixed 1:1 and subjected to acetone precipitation. Protein pellets were dissolved in rehydration buffer and resolved on SDS-PAGE or two-dimensional electrophoresis (2-DE; see details below).

Fluorescence images were acquired using the Fuji 5000 or Typhoon 9400 imager. Fluorescence intensity of spots on 2-DE gels was quantified using the SameSpots software (TotalLab, UK, version 4.5) [31]. Spots consistently exhibiting average fold difference >1.3 (*P* <0.05) between control and treatment samples on three replicate gels were selected and excised on zinc-stained gels. Those gel samples were then subjected to protein trypsin digestion (see below) into peptides for protein identification using liquid chromatography coupled to tandem mass spectrometry (LC-MS/MS). *S*-Nitrosylation of the selected proteins was validated using the BST method.

### 2-DE

Protein samples (400 μg) were dissolved in 450 μL rehydration buffer (8 M Urea, 4% CHAPS, 20 mM DTT, 0.5% IPG buffer, pH range 3–10) and resolved on a 24-cm IPG strip (pH 3–10). Strips were incubated with equilibration buffer (50 mM Tris-Cl, pH 8.0, 6 M urea, 2% SDS, and 30% glycerol) containing 1% DTT or 2.5% iodoacetamide sequentially for 15 minutes. Proteins on the strips were further resolved by 12% SDS-PAGE. All gels were run at 1 Watt/gel overnight in the dark.

### SDS-PAGE gel staining and Western blotting

Proteins were separated on 10% or 12% SDS-PAGE. For zinc-reverse staining, gels were washed briefly with Milli-Q water and then incubated in an imidazole-SDS solution (200 mM imidazole, 0.1% SDS) for 5 minutes. After brief washing with Milli-Q water, gels were developed with a 200 mM zinc sulfate solution. Within 20 seconds, the gel background became white, while protein spots remained opaque. Development was stopped by discarding the zinc sulfate solution and immersing gels into a large quantity of Milli-Q water. Developed gels were stored in Milli-Q water prior to image acquisition. Silver staining was performed with a ProteoSilver™ Silver Stain Kit (Sigma-Aldrich). For Western blotting, a nitrocellulose membrane was first incubated in PBS-T buffer (0.1% Tween-20 in PBS, pH 7.4) containing 5% nonfat milk at room temperature for 1 hour and then incubated with primary antibody at 4°C overnight. After washing with PBS-T, the membrane was incubated with a secondary antibody at room temperature for 1 hour. Immuno-reactive bands were detected using a SuperSignal West Pico chemiluminescence detection system (Pierce).

### In-gel trypsin digestion of proteins

Selected zinc-stained 2-DE gel spots of approximate 1 mm diameter were manually excised, washed with 2% citric acid twice to remove zinc-staining reagents, and then placed into a 96-well plate for in-gel trypsin digestion, as previously described [7,16]. Briefly, in-gel digestion of proteins was carried out using 0.2 μg/μL sequencing-grade modified trypsin (Promega). After discarding the excess solution, gel slices were incubated with 30 μL of 50 mM ammonium bicarbonate at 37°C for 16 hours. The supernatant of the digested peptides was transferred to a clean 96-well plate. Gel slices were washed twice for 10 minutes each with 25 μL of extraction solution containing 50% acetonitrile and 1% trifluoroacetic acid. Extracted peptides were pooled and lyophilized by centrifugal evaporation.

### Protein identification by mass spectrometry

#### ***Q-TOF***

An Agilent 6520 Q-TOF with HPLC-Chip Cube electrospray ionization source was used for protein identification. Specifically, the digested peptides were re-suspended in 8 μL formic acid (1% in water), and a portion of the digests (5 μL) was loaded onto an Agilent chip LC integrated enrichment column followed by a 43 mm × 75 μm analytical column packed with Zorbax C18 (300 A, 5-μm particles). Peptides were eluted from the analytical column in a continuous LC gradient of 10–40% B in 10 minutes with a flow rate of 600 nL/minute (A: 0.1% formic acid in 18 Mohm water, B: 99.9% acetonitrile, 0.1% formic acid). The data were acquired in the positive ion mode at 2 spectra/sec for both MS and MS/MS. The top five peptides in each cycle (3.1 sec) with absolute threshold over 2,500 counts or relative abundance over 0.01% were selected for MS/MS acquisition. The peptides were excluded after one spectrum and released from exclusion list after 0.25 minutes, and the ions of charge state +1 and unknown were ignored. The collision energy for each peptide was automatically calculated using the formula of (slope of 3.25) × m/z/ 100 + offset of 2.

The acquired data were extracted using the Agilent Qualitative Analysis program with the following parameters: retention time window for peptide mass extraction 0.25 minutes, MS/MS fragment signal threshold of 50 counts, match tolerance (for different charge states of the same peptide) of 0.05 m/z, limit to the 3,000 most-abundant peptides. The extracted data were exported as MASCOT generic format files and then searched using MASCOT against the IPI-mouseV3.80 database downloaded on November 20, 2012 with 54,285 protein entries. The mass accuracy was set as 25 ppm, and allowed one missed cleavage for trypsin. Carbamidomethylation of cysteine was selected as a static modification, and oxidized methionine was specified as a variable modification. MASCOT searches were conducted using the automatic decoy search utility which implements a reversed decoy database search strategy to calculate peptide false discovery rate. The identified proteins were filtered by the criteria of sequence coverage >5%, peptide false discovery rate <10%, and matched peptides per protein ≥1.

#### ***LTQ Orbitrap-XL***

The digested peptides were analyzed using LTQ Orbitrap-XL as previously described [32], with the following modifications: a short LC-MS/MS gradient (ramp to 0–40% B over 15 minutes) was used, and data were searched against the IPI-mouseV3.80 database using Sorcerer-Sequest searching engine (Sage-N Research, San Jose, CA, USA). Following a high-resolution (30,000 res, profile) Fourier Transform MS scan of the eluting peptides (300–2,000 m/z range), in each cycle, the 9 most abundant peptides (reject trypsin autolysis ions) were subjected to collision-induced dissociation peptide fragmentation (>1,000 counts, NCE of 35%, centroid). Automatic gain control was targeted at 3e^5^ and 3e^4^ with maximal injection time of 500 ms and 800 ms for a full scan and MS/MS scan, respectively. Data across a total of 35 minutes of elution were collected. The same criteria as described above in Q-TOF part were applied to filter the identified proteins.

### Pathway analysis and functional annotation

Given the identified differentially *S*-nitrosylated proteins, molecular and cellular functions, canonical pathway, and protein networks were predicted using Ingenuity Pathway Analysis (IPA). Our in-house MULTICOM-PDCN software [33,34] was used to predict protein subcellular locations by searching the Swiss-Prot database [35].

## Results

### Detection of SNO-proteins by the NitroDIGE method

**Figure 1:**
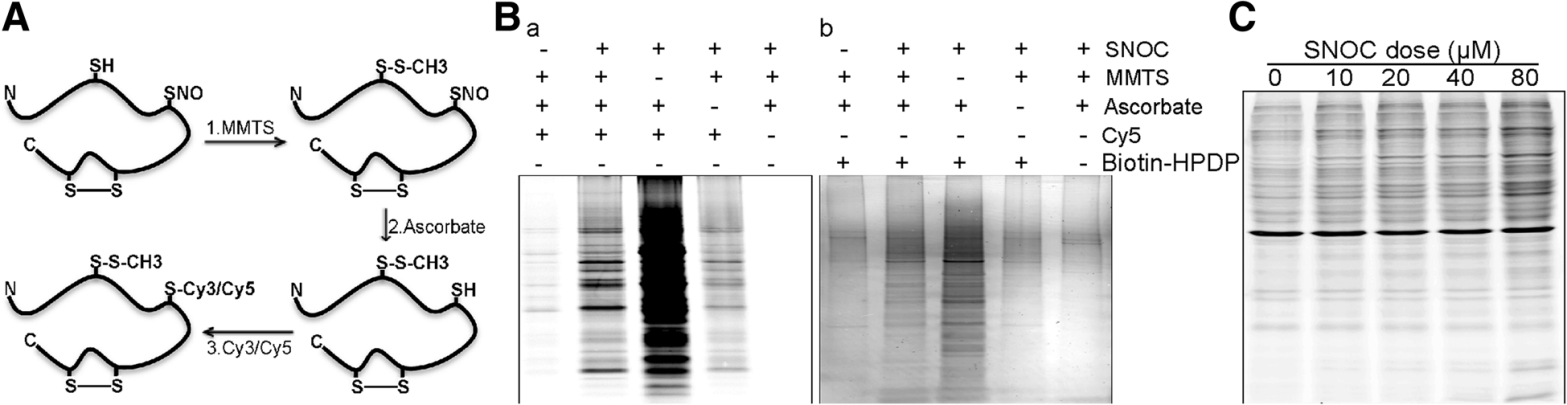
**Development of NitroDIGE to detect SNO**-**proteins. (A)** Schematic showing the NitroDIGE method for labeling of *S*-nitrosylated cysteine thiols. A hypothetical protein is indicated with cysteines in the free thiol, disulfide, or nitrosothiol conformation. Free thiols are first blocked with MMTS. Ascorbate selectively releases NO from *S*-nitrosylated cysteine thiols. The fluorescent thiol-reactive CyDye™ (Cy3 or Cy5) reacts with NO-released thiols to form stable fluorescent complexes. **(B)** The specificity and sensitivity of NitroDIGE. **(a)** Cell lysates (20 μg) were treated with 200 μM SNOC, and SNO-proteins were analyzed by NitroDIGE; 5 μg of proteins from each NitroDIGE-labeled sample were separated by SDS-PAGE and Cy5 fluorescence signals were collected. Control omitting SNOC, MMTS, ascorbate, or Cy5 demonstrated that CyDye™ specifically labeled SNO-proteins. **(b)** In the BST, 250 μg of SNO-proteins were labeled with Biotin-HPDP, pulled down by avidin-agarose, and visualized by silver staining. **(C)** BV-2 cell lysates were exposed to different doses of SNOC as a test of sensitivity. The NitroDIGE method detected SNO-proteins in cell lysates treated with as low as 10 μM SNOC.

In this study, we developed a NitroDIGE method for the determination of redox-based protein *S*-nitrosylation. The NitroDIGE method uses the same blocking and reduction steps as the previously established BST, but the specific thiol linker Biotin-HPDP is replaced with the irreversible fluorescence-based thiol reactive reagents, maleimide-linked dyes (Cy3 and Cy5) [15], which label the nascent thiols reduced from *S*-nitrosocysteines by ascorbate (Figure 1A). More specifically, free thiols were blocked by methylthiolation with MMTS, and then the excessive un-labeled MMTS was removed by acetone precipitation. Nitrosothiols on SNO-proteins were selectively reduced with ascorbate and then reacted with the fluorescence (Cy3 or Cy5)-tagged thiol linkers, forming stable fluorescence-tagged complexes.

To evaluate the specificity of maleimide-linked dyes in labeling ascorbate-reduced cysteines, 20 μg of BV-2 cell lysates were exposed to a physiological NO donor, 200 μM SNOC, at room temperature for 30 minutes, and then SNO-proteins were labeled with CyDye™ as described above; 5 μg of the labeled cell lysate samples was resolved by SDS-PAGE, and SNO-proteins were visualized using a Fuji 5000 fluorescence scanner (Figure 1B,a). Various controls by omitting SNOC, MMTS, or ascorbate verified the specificity of NitroDIGE labeling. For comparison, BST using Biotin-HPDP to label SNO-proteins instead of CyDye™ (Figure 1B,b) was conducted. NitroDIGE and BST resulted in a similar overall pattern of SNO-proteins, but a larger amount of starting material was needed for the latter (250 μg protein for BST versus 20 μg for NitroDIGE).

Sensitivity is a technical challenge in detecting *in vivo* SNO-proteins, since cysteine residues account for approximately 2.3% in the human proteome, and protein *S*-nitrosylation is reversible serving as molecular switch to regulate various biological processes in the cell [3,36]. We tested NitroDIGE labeling sensitivity in response to different concentrations of NO by exposing BV-2 cell lysates to low doses (0–80 μM) of SNOC under physiological conditions. Our results showed that the NitroDIGE method detected protein *S*-nitrosylation even in the presence of 10 μM SNOC (Figure 1C). We conclude that NitroDIGE labeling is specific for protein *S*-nitrosylation and displays high sensitivity.

### Identification of SNO-proteins in BV-2 cells *ex vivo* exposed to NO donor

**Figure 2:**
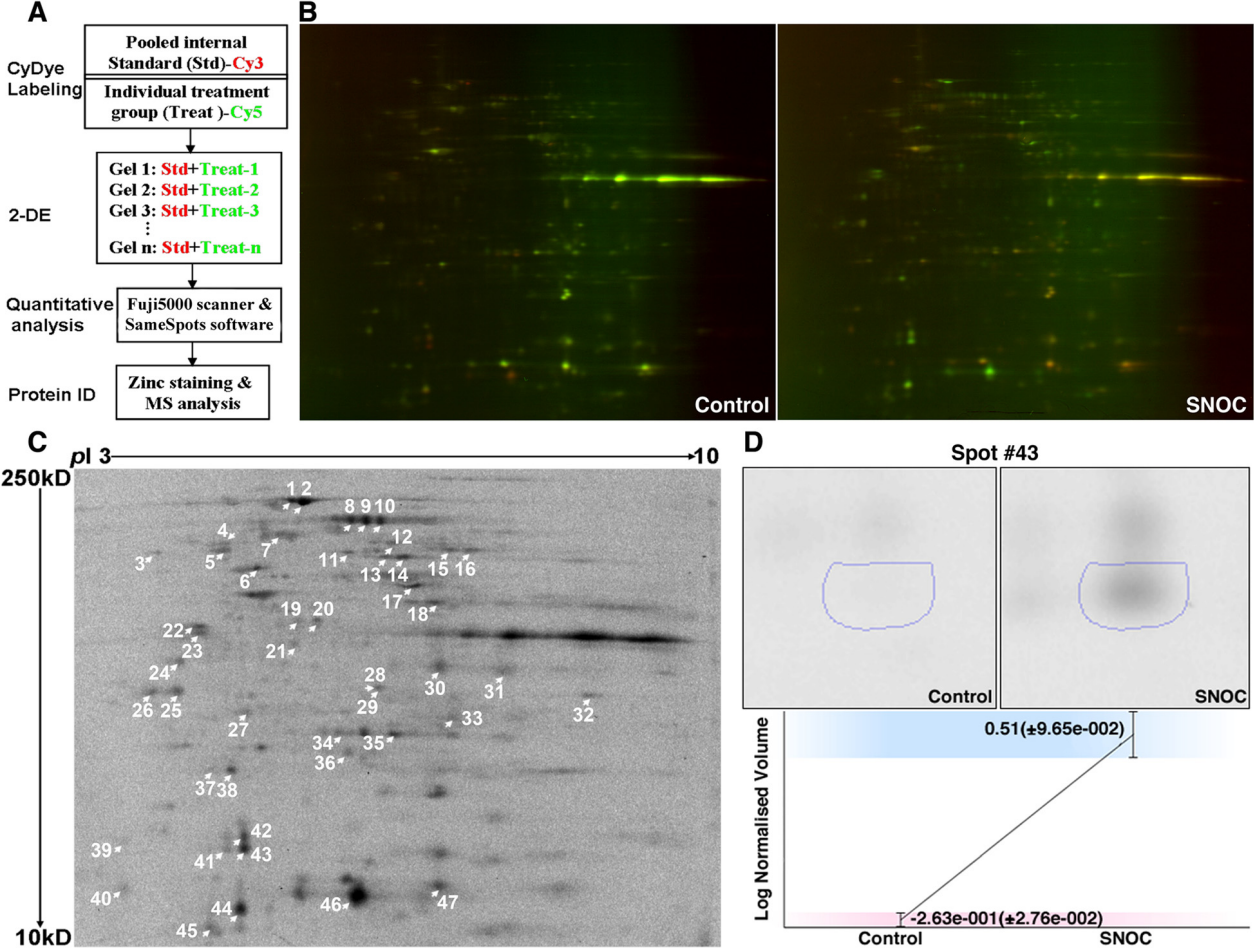
**Identification and quantification of SNOC**-**induced protein *S****-***nitrosylation in BV**-**2 cells by NitroDIGE. (A)** Workflow of NitroDIGE to identify and quantify SNO-proteins. Pooled internal standard and individual samples are labeled with Cy3 and Cy5, respectively, and subjected to 2-DE. 2-DE gel fluorescence is detected using a Fuji 5000 scanner and quantified by the SameSpots software. On a corresponding zinc-staining gel, selected fluorescence intensity-differential spots are excised for MS analysis. **(B)** NitroDIGE analysis of protein *S*-nitrosylation in *ex vivo* SNOC-treated BV-2 cells. Following the work flow above, untreated control and SNOC-treated samples in biological triplicate were labeled with CyDye™ and resolved on six different gels. A representative gel from each group is shown. **(C)** Quantitative analysis with the SameSpots software revealed 47 spots with significant fluorescence intensity changes between SNOC-treated and untreated control samples (fold change >1.3, *P* <0.05). **(D)** Quantification results for spot #43 are displayed as an example.

We next combined the NitroDIGE labeling assay with 2-DE and MS techniques to profile SNO-proteins (Figure 2A). First, an internal standard was established by pooling from all of the samples in equal amounts. One can employ either Cy3 or Cy5 to label the pooled internal standard (we used Cy3 labeling in this study), and use the other dye to label individual samples. After labeling, equal amounts of the pooled internal standard and individual treatment samples were mixed and subjected to 2-DE, fluorescence scanning, and quantitative analysis by the SameSpots software. Spots on 2-DE gels exhibiting an average fold difference >1.3 (*P* <0.05) between control and treatment samples were considered significantly different. The corresponding spots (SNO-proteins) on a zinc staining gel were excised for protein identification by LC-MS/MS analysis. In a pilot study of the CyDye™ fluorescence dyes, we conducted Cy3 and Cy5 dye-swap labeling to confirm equality and specificity of these two dyes. Compared to the control, samples exposed to SNOC showed a significant signal increase using either fluorescence dye (data not shown).

To detect protein *S*-nitrosylation relevant to neuroinflammation, we exposed BV-2 microglial cells to 20 μM of the NO donor SNOC for 30 minutes and then analyzed the cell lysates by the NitroDIGE method. Resulting representative 2-DE gels are shown as Figure 2B. Comparison between the untreated and the SNOC-treated samples revealed 47 spots on the 2-DE gel that were significantly different (fold change >1.3, *P* <0.05) in spot fluorescence intensity (Figure 2C). As an example, the quantitative result for spot #43 is shown in Figure 2D. The *S*-nitrosylation level of this spot increased 6-fold after exposure to SNOC. With MS/MS analysis, we identified 67 unique proteins from the 47 spots with differential fluorescence intensities (Additional file 1: Table S1). We submitted these proteins for pathway analysis using IPA, and the top 10 pathways are shown in Additional file 2: Figure S1.

### Identification of SNO-proteins in LPS-activated BV-2 cells

**Figure 3:**
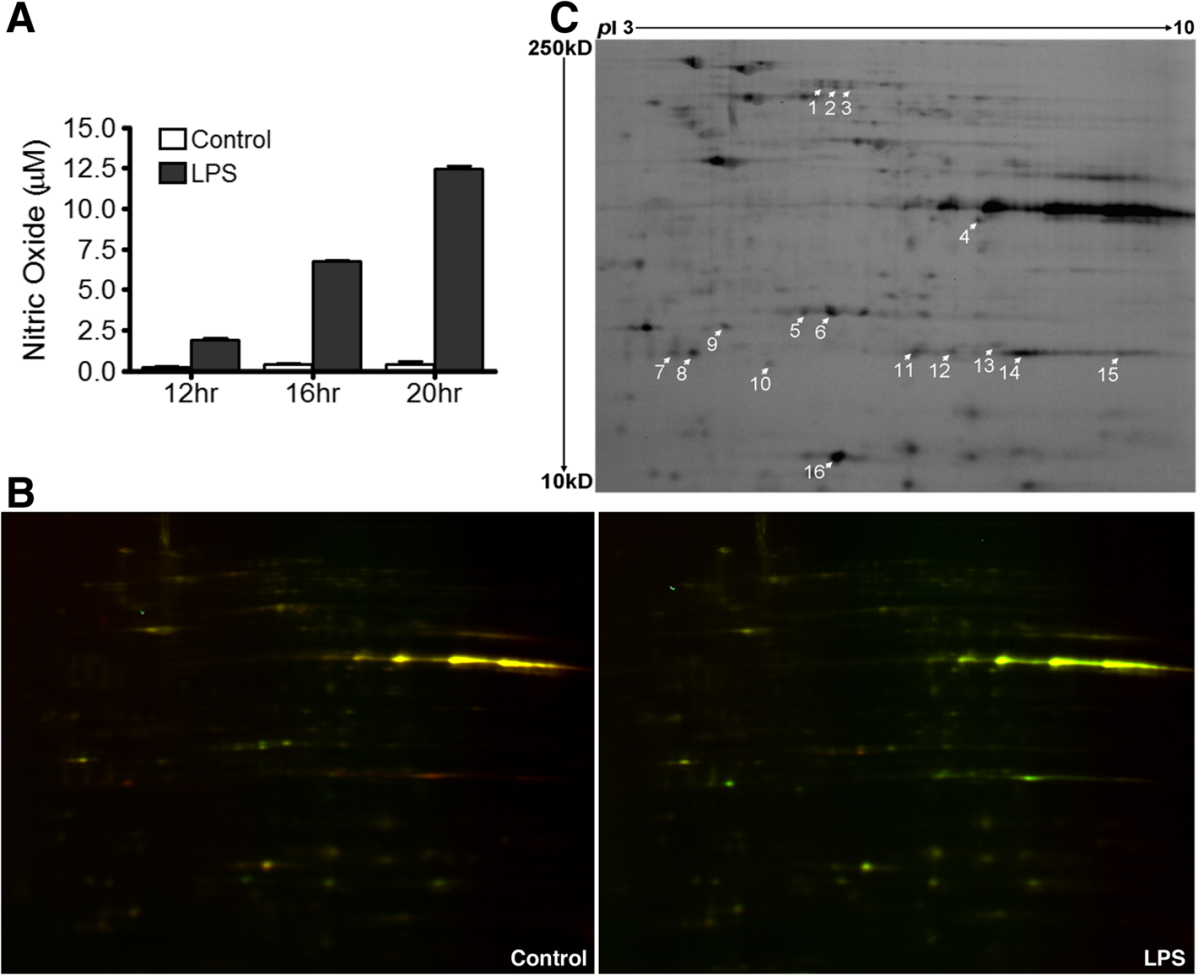
**LPS**-**induced protein *S***-**nitrosylation in BV**-**2 cells. (A)** LPS-induced NO production. BV-2 cells were treated or untreated with 100 ng/mL LPS for 12, 16, or 20 hours. A Griess assay indicated NO production was elevated in the conditioned medium as the LPS incubation time increased. **(B)** NitroDIGE analysis of protein *S*-nitrosylation in BV-2 cells treated with LPS for 20 hours. Compared to untreated control, more green spots were seen from LPS-treated sample on the 2-DE gel. **(C)** Quantitative analysis revealed a total of 16 spots with significant *S*-nitrosylation level changes (fold change >1.3, *P* <0.05), from which 13 unique proteins were identified (Additional file 3: Table S2).

We further investigated protein *S*-nitrosylation in microglial cells by using 100 ng/mL LPS, a condition known to induce proinflammatory responses with increased NO production. A Griess assay indicated that after LPS treatment, NO concentrations in BV-2 cells significantly increased in a time-dependent manner (Figure 3A) and reached to 10 to 20 μM after 20 hours of treatment. We processed the BV-2 cells untreated or treated with LPS for 20 hours for further NitroDIGE analysis. Cy3 was employed to label the pooled internal standard and Cy5 was utilized to label each of the individual samples. More green spots were observed on the gels from LPS-treated samples compared to the untreated control (Figure 3B), suggesting *S*-nitrosylation levels of some proteins were up-regulated. NitroDIGE analysis identified 13 unique proteins from 16 differentially *S*-nitrosylated protein spots (fold change >1.3, *P* <0.05) from LPS-stimulated BV-2 cells (Figure 3C). Full protein identification data are listed in Additional file 3: Table S2, and the top 10 pathways by IPA are shown in Additional file 4: Figure S2.

### Effect of EGCG on LPS-induced protein *S*-nitrosylation in BV-2 cells

NitroDIGE is also useful in the analysis of multiple group samples in the study of botanical compounds. EGCG is a phenolic compound found in green tea and is known to exert antioxidant effects. To determine whether nitrosative stress-induced protein *S*-nitrosylation could be modulated by EGCG, as well as to understand the mode(s) of action of EGCG on proinflammatory responses in microglial cells, we employed NitroDIGE to assess the effects of EGCG on LPS-induced microglial activation in BV-2 cells.

**Figure 4:**
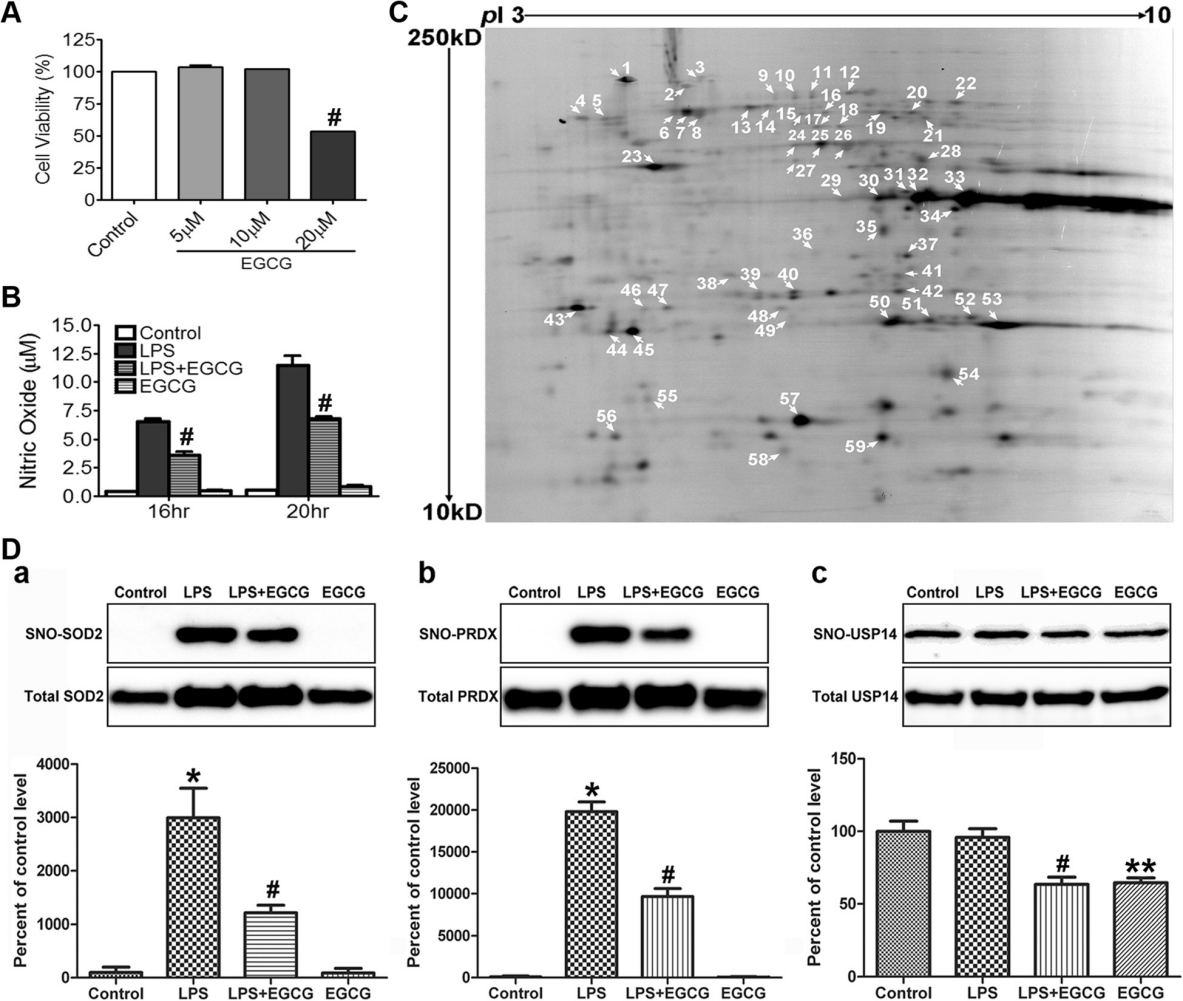
**Effect of EGCG on LPS**-**induced protein *S***-**nitrosylation in BV**-**2 cells. (A)** Dose titration for EGCG. BV-2 cells were treated with 0, 5, 10, and 20 μM EGCG for 20 hours and cell viability was assessed by a MTT assay (# *P* <0.01, control vs. 20 μM EGCG, n = 3). **(B)** Administration of EGCG (10 μM) 1 hour prior to LPS (100 ng/mL) exposure inhibited NO production in BV-2 cells (# *P* <0.01, LPS vs. LPS + EGCG, n = 3). **(C)** A total of 59 differentially *S*-nitrosylated protein spots were detected by NitroDIGE analysis (fold change > 1.3, *P* <0.05, LPS vs. LPS + EGCG, n = 3). **(D)** Seventy-eight proteins were identified from the above spots by LC-MS/MS, and SOD2 (a), PRDX (b), and USP14(c) were selected for validation by the BST method. After Biotin-HPDP labeling and biotin affinity pull-down, individual protein Western blotting was performed. The amount of SNO-proteins was quantified by a densitometer, normalized to total proteins, and expressed as percentage of untreated controls. Data are means ± SEM (n = 3); * *P* <0.05, untreated vs. LPS; # *P* <0.05, LPS vs. LPS + EGCG; ** *P* <0.05, untreated vs. EGCG. The results showed the down-regulation of *S*-nitrosylation levels of these proteins in “LPS + EGCG” compared to LPS-treated samples.

We first used the MTT assay to show that BV-2 cell viability was not affected by treatment with 5 or 10 μM EGCG for 20 hours. However, cells exposed to higher concentrations of EGCG (20 μM) displayed reduced viability (Figure 4A). Thus, we chose 10 μM to assess the effect of EGCG treatment on nitrosative stress and protein *S*-nitrosylation in microglial cells. A Griess assay indicated that administration of 10 μM EGCG for 1 hour prior to LPS exposure significantly inhibited LPS-induced NO production in BV-2 cells (Figure 4B).

The samples (untreated, LPS-treated, EGCG-treated, LPS + EGCG-treated) were then analyzed by NitroDIGE. SameSpots analysis revealed 59 spots on the 2-DE gels with significant changes (fold change >1.3, *P* <0.05) in *S*-nitrosylation levels when comparing between LPS-treated and LPS + EGCG-treated samples (Figure 4C). EGCG alone did not have any significant impact on protein *S*-nitrosylation (data not shown). In total, 78 unique proteins were identified from these spots (Additional file 5: Table S3). Among these identified SNO-proteins, PRDX, SOD2, and USP14 were tested for validation using the BST method (Figure 4D). After avidin-agarose pull-down, individual protein Western blot results showed *S*-nitrosylation levels of PRDX and SOD2 increased after LPS exposure compared to untreated control, but were attenuated by addition of EGCG. For USP14, LPS did not induce any change, but EGCG was able to down-regulate its *S*-nitrosylation level, which confirms our NitroDIGE results.

### Pathways and functional analysis

**Figure 5:**
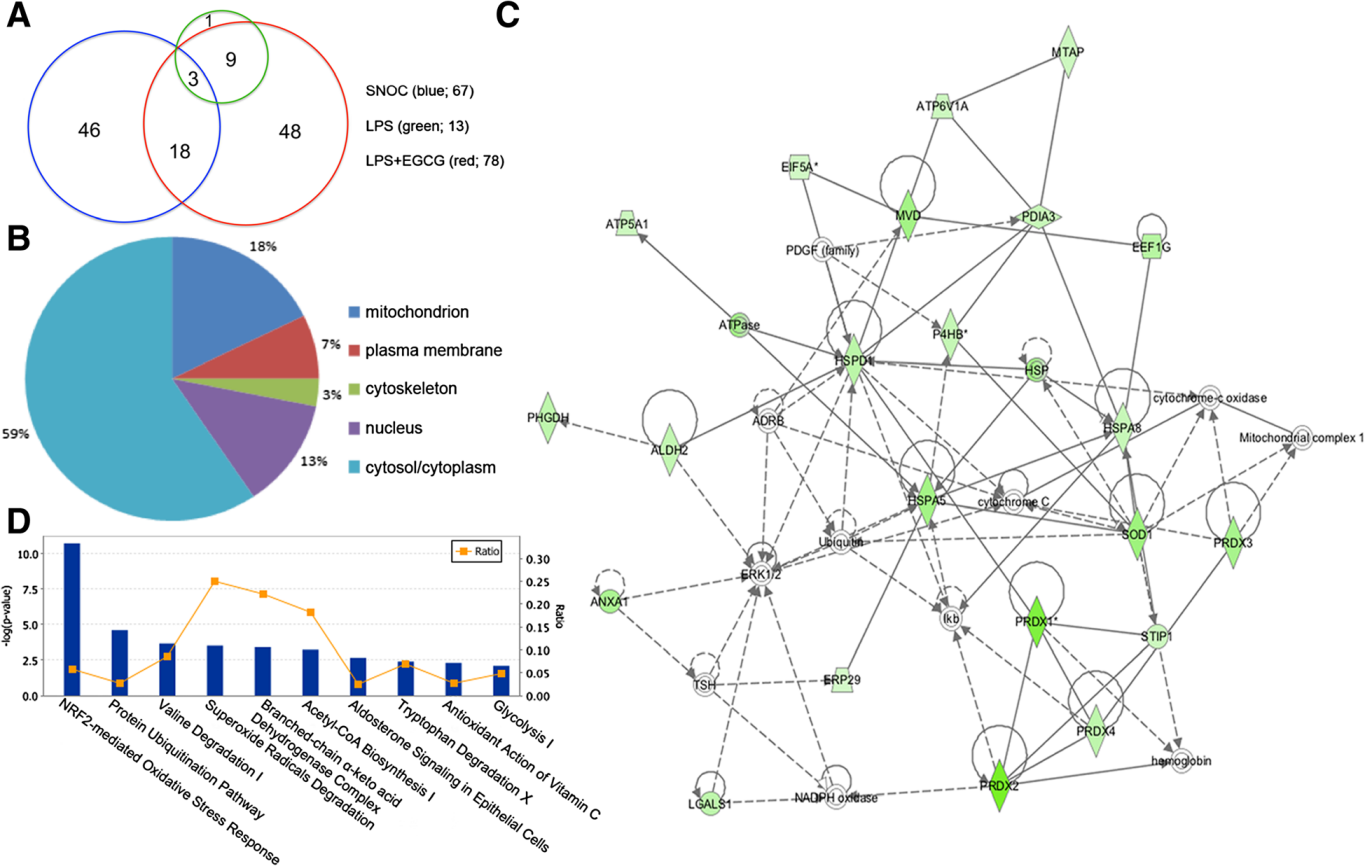
**Functional annotation and pathway analysis. (A)** Three subsets of SNO-proteins with overlaps in between were identified from different treatments in this study. There are three common proteins shared by the three data sets, including TCP1, ISYNA1, and PRDX2. **(B)** Cellular location of the 78 SNO-proteins responding to EGCG treatment in LPS-stimulated BV-2 cells. **(C)** The top protein interaction network associated with EGCG-treatment in LPS-stimulated BV-2 cells was predicted by IPA and presented. Twenty-four SNO-proteins are involved in this network. The intensity of green color indicates the level of down-regulation. **(D)** Top 10 IPA canonical pathways targeted by EGCG in LPS-stimulated BV-2 cells.

In this study, we identified 67, 13, and 78 SNO-proteins in response to SNOC, LPS, and LPS + EGCG treatments, respectively (Figure 5A). There were three common SNO-proteins, TCP1, ISYNA1, and PRDX2, found between SNOC and LPS treatments, suggesting distinct NO signaling pathways were triggered under these two conditions (Additional file 2: Figure S1 and Additional file 4: Figure S2). Most of the SNO-proteins (12 out of 13) found in the LPS-treated sample were also identified in the LPS + EGCG group (Figure 5A), indicating that EGCG sufficiently attenuates the effects of LPS on protein *S*-nitrosylation. Proteins in the LPS + EGCG group had 21 proteins in common with the SNOC-treated sample and 57 other proteins.

In order to understand the mechanisms for EGCG to down-regulate *S*-nitrosylation under LPS-stimulated microglial activation, the identified SNO-proteins were subjected to functional annotation using IPA and the in-house MULTICOM-PDCN software. MULTICOM-PDCN analysis showed that the 78 proteins responding to EGCG are mainly located in the cytoplasm (59%), mitochondrion (18%), and nucleus (13%) (Figure 5B). They mainly play roles in immunological disease, inflammatory disease, and neurological disease as predicted by IPA. The top protein network associated with immunological disease and inflammatory disease is shown as Figure 5C; 24 out of the 78 SNO-proteins are involved in this network. IPA annotation of the top canonical pathways revealed that the action of EGCG significantly involved nuclear factor erythroid 2-related factor 2 (Nrf2)-mediated oxidative stress response, protein ubiquitination, valine degradation, superoxide radical degradation, and branched-chain α-keto acid dehydrogenase complex (Figure 5D). Molecule targets identified in these pathways are listed in Table 1.

**Table 1:** **IPA annotation of the top canonical pathways altered by EGCG in LPS**-**stimulated BV**-**2 cells**

**Name**	***P *** **value**	**Ratio**	**Molecules**
NRF2-mediated oxidative stress response	1.9E-11	11/192 (0.057)	ACTB, AKR1A1, CLPP, ERP29, GSTO1, HMOX1, PRDX1, SOD1, SOD2, STIP1, USP14
Protein ubiquitination pathway	2.6E-05	7/268 (0.026)	HSPA5, HSPA8, HSPD1, PSMA1, PSMB2, PSMD7, USP14
Valine degradation I	0.00022	3/35 (0.086)	DBT, DLD, HIBCH
Superoxide radicals degradation	0.00031	2/8 (0.25)	SOD1, SOD2
Branched-chain α-Keto acid dehydrogenase complex	0.0004	2/9 (0.222)	DBT, DLD

Notes: A ratio indicates the number of identified differentially *S*-nitrosylated proteins map to the pathway divided by the total number of proteins that exist in the pathway.

We then asked what underlying molecular and cellular functions are associated with the mode of action of EGCG in LPS-induced microglial activation. IPA annotation with the identified proteins responding to EGCG revealed that EGCG was significantly involved in free radical scavenging (9 proteins), PTM (15 proteins), protein folding (7 proteins), nucleic acid metabolism (17 proteins), and small molecule biochemistry (34 proteins) (see details in Table 2). Thus, these findings suggest that EGCG exhibited multi-modal action by alleviating NO production and further protein *S*-nitrosylation under microglial activation.

**Table 2:** **IPA annotation of molecular and cellular functions for the action of EGCG in LPS**-**stimulated BV**-**2 cells**

**Name**	***P *** **value**	**# Molecules**	**Molecules**
Free radical scavenging	1.56E-09 – 1.38E-02	9	ACTB, ALDH2, ANXA1, HMOX1, PRDX1, PRDX2, PRDX3, SOD1, SOD2
Post-translational modification	9.96E-09 – 1.01E-02	15	ACADL, ALDH2, ERP29, GLUD1, HMOX1, HPRT1, HSPA5, HSPA8, HSPD1, IMPDH2, LRPAP1, P4HB, SOD1, SOD2, TCP1
Protein folding	9.96E-09 – 3.37E-03	7	ERP29, HSPA5, HSPA8, HSPD1, LRPAP1, P4HB, TCP1
Nucleic acid metabolism	1.78E-07 – 1.67E-02	17	ALDH2, ATIC, ATP5A1, CMPK2, HMOX1, HPRT1, HSPA5, HSPA8, HSPD1, IMPDH2, MTAP, PKM, RUVBL1, SOD1, SOD2, TALDO1, UMPS
Small molecule biochemistry	4.34E-07 – 1.67E-02	34	ACADL, AKR1A1, ALDH2, ANXA1, ATIC, ATP5A1, CMPK2, DLD, FABP5, GLUD1, GNPDA1, GSTO1, HMOX1, HPRT1, HSPA5, HSPA8, HSPD1, IMPDH2, LGALS1, MTAP, MVD, P4HB, PDIA3, PHGDH, PKM, PRDX1, PRDX2, PRDX3, RUVBL1, SOD1, SOD2, TALDO1, TIMM50, UMPS

## Discussion

Increasing evidence indicates that protein *S*-nitrosylation, a reversible post-translation cysteine modification process, plays a critical role in NO signaling pathways [3,36,37]. Recent studies have been carried out to identify SNO-proteins and their functions in cellular signaling and in various disease states. Here, we demonstrated that NitroDIGE is a relatively low-cost (compared to isotopic labeling with LC/MS shot-gun analysis) proteomics screening strategy for identifying proteins modified by *S*-nitrosylation under nitrosative stress and effects of botanical compounds on protein *S*-nitrosylation. More importantly, this method, combined with the CyDye™ switch labeling of *S*-nitrosylated cysteines, 2-D DIGE and LC-MS/MS analysis, is sensitive, quantitative, and utilizes a smaller sample size. In this study, we have successfully identified 67 and 13 proteins as putative targets for *S*-nitrosylation in BV-2 cells after exposure to SNOC and LPS, respectively. In addition, we revealed the multi-modal action of EGCG in LPS-stimulated BV-2 microglial cells.

The CyDye™ DIGE Fluor dyes were used for the NitroDIGE method rather than the Biotin-HPDP as used in the BST method. These dyes specifically bind to cysteine thiol groups and form bonds that are stable upon reduction by reagents such as DTT and tris(2-carboxyethyl)phosphine. Thus, thiol-labeling is not lost during sample analyses, such as electrophoresis. After CyDye™ labeling, SNO-proteins are easily detected with a fluorescence scanner (e.g., the Fuji 5000) and no additional staining or immunoblotting is needed. In previous reports, labeling with CyDye™ or other fluorescence dyes has been used to compare protein *S*-nitrosylation in two different samples within one gel [38-41]. To analyze comprehensive samples, we introduced an internal standard in the 2-D DIGE experimental design. The internal standard is pooled from every sample in equal amounts, labeled with either Cy3 or Cy5, and then run on each gel together with an experimental sample labeled with the other CyDye™. *S*-Nitrosylation levels of multiple samples with biological replicates can be compared at the same time on different gels, reducing system variability. Therefore, the NitroDIGE approach can facilitate multi-group studies of protein *S*-nitrosylation and differentiate changes under various treatment conditions.

Many neurological disorders involve nitrosative stress and proinflammatory responses with activation of microglial cells [42-44]. A comprehensive investigation of the proteins affected by *S*-nitrosylation could enhance our understanding of molecular mechanisms underlying NO signaling in activated microglial cells. In this study, NitroDIGE analysis of SNOC/LPS-stimulated BV-2 cells revealed a group of proteins as putative *S*-nitrosylation targets (Additional file 1: Table S1 and Additional file 3: Table S2). These proteins are linked to multi-modal functions, e.g., protein degradation, protein folding, stress responses, free radical scavenging, cell death and survival, and PTM. Distinct canonical pathways are involved in SNOC and LPS stimulation, as predicted by IPA (Additional file 2: Figure S1 and Additional file 4: Figure S2). One group of proteins identified is the redox system of PRDXs, including PRDX1, PRDX2, PRDX3, and PRDX4, which are members of a family of antioxidant enzymes that reduce H_2_O_2_ by other hydroperoxides and peroxynitrite generated in cells under physiological and pathological conditions [45]. Our findings are consistent with the previous report showing that the activity of such redox-sensitive proteins is modulated by *S*-nitrosylation [12].

Evidence suggests that EGCG, the major component of the green tea polyphenols, is an efficient scavenger of oxygen and nitrogen radical species [46,47], and has protective effects against nitrosative stress and neuronal cell death in a variety of neurodegenerative disorders including Parkinson’s disease, Alzheimer’s disease, Huntington’s disease, amyotrophic lateral sclerosis, and stroke [48-50]. Various molecular signaling pathways are implicated in EGCG-induced neuroprotection [51], such as mitogen-activated protein kinases [52], protein kinase C [53,54], phosphatidylinositol-3-kinase/Akt signaling pathways [55-57], and regulation of antioxidant response genes and proteins [58-60]. In LPS-stimulated BV-2 cells, EGCG attenuates NO production via the down-regulation of inducible NO synthase [25,61]. Here, utilizing the NitroDIGE method, we further investigated the effect of EGCG on protein *S*-nitrosylation under microglial activation. We found that EGCG treatment decreased *S*-nitrosylation levels of 78 proteins (Additional file 5: Table S3). These proteins mainly function in free radical scavenging, PTM, and protein folding (Table 2), and are associated with immunological disease and inflammatory disease (Figure 5C). Changes in the levels of *S*-nitrosylation of these proteins may regulate their activities and thus affect their functions in diverse biological processes, as demonstrated in several studies [5-9]. A previous study reported that EGCG regulated the activity of striatal SOD in MPTP-treated mice [62]. Indeed, the *S*-nitrosylation level of SOD was found to be significantly down-regulated by EGCG in this study, making *S*-nitrosylation a potent candidate mechanism for regulation of SOD or other proteins. Furthermore, Nrf2-mediated oxidative stress response, the primary cellular defense against oxidative stress [63], was predicted by IPA to be the top pathway responding to EGCG treatment in LPS-stimulated BV-2 cells (Figure 5D). Eleven proteins from this pathway were found undergoing *S*-nitrosylation alteration by EGCG (Table 1), and some of them were validated by the BST method (Figure 4D). In the Nrf2-mediated pathway, under conditions of increased nitrosative stress, Nrf2 is activated and hence triggers antioxidant response element-driven expression of detoxification and antioxidant genes [64,65]. In human breast epithelial (MCF10A) cells, EGCG was reported to regulate Nrf2-mediated expressions of several antioxidant enzymes, including glutamate-cysteine ligase, SOD, and HMOX1 [66]. Attenuation of protein *S*-nitrosylation by EGCG in Nrf2 and other signaling pathways may have profound impact on nitrosative defense, implying another mode of action for EGCG.

## Conclusions

Taken together, our application of the NitroDIGE method demonstrates that it is a promising and powerful tool to profile SNO-proteins under a variety of conditions. Our findings provide molecular mechanistic insights into NO signaling in microglia and EGCG’s multi-modal action upon protein *S*-nitrosylation, as well as on nitrosative stress, under microglial activation.

## Abbreviations

2-D DIGE: Two-dimensional differential in-gel electrophoresis; 2-DE: Two-dimensional electrophoresis; Biotin-HPDP: N-[6-(biotinamido)hexyl]-3’-(2’-pyridyldithio) propionamide; BST: Biotin switch technique; DMEM: Dulbecco’s modified Eagle’s medium; DTT: Dithiothreitol; EGCG: Epigallocatechin-3-gallate; FBS: Fetal bovine serum; HEN: Hepes/EDTA/Neocuproine; IPA: Ingenuity pathway analysis; LC-MS/MS: Liquid chromatography coupled to tandem mass spectrometry; LPS: Lipopolysaccharides; MMTS: Methyl methanethiosulfonate; MTT: [3-(4,5-dimethylthiazol-2-yl)-2,5-diphenyl-2H-tetrazolium bromide; NO: Nitric oxide; Nrf2: Nuclear factor erythroid 2-related factor 2; PRDX: Peroxiredoxins; PTM: Post-translational modification; SNO-proteins: *S*-Nitrosylated proteins; SNOC: *S*-Nitrosocysteine; SOD2: Superoxide dismutase 2; USP14: Ubiquitin carboxyl-terminal hydrolase 14.

## Competing interests

The authors declare that they have no competing interests.

## Authors’ contributions

ZG conceived and designed the project; GYS provided BV-2 cells; ZQ, FM, and FW performed experiments; ZQ, FM, HZ, JL, QW, and ZG analyzed data; ZQ, FM, and ZG wrote the manuscript with significant input from JC, CMG, DBL, GYS, and SL. All authors have read and approved the final manuscript.

## Supplementary Material

Additional file 1: Table S1 LC-MS/MS identification of SNO-proteins in BV-2 cells exposed to SNOC. Click here for file

Additional file 2: Figure S1 IPA analysis of protein *S*-nitrosylation in *ex vivo* SNOC-treated BV-2 cells. A total of 67 SNO-proteins were identified from SNOC-treated BV-2 cells and the top 10 canonical pathways involved by these proteins were predicted by IPA analysis. Click here for file

Additional file 3: Table S2 LC-MS/MS identification of SNO-proteins in LPS-stimulated BV-2 cells. Click here for file

Additional file 4: Figure S2 IPA analysis of protein *S*-nitrosylation in LPS-stimulated BV-2 cells. In total, 13 SNO-proteins were identified from LPS-stimulated BV-2 microglial cells, and the top 10 canonical pathways participated by these proteins were predicted by IPA. Click here for file

Additional file 5: Table S3 LC-MS/MS identification of SNO-proteins responding to EGCG in LPS-stimulated BV-2 cells. Click here for file
