# Supplementary figures and images for: NitroDIGE analysis reveals inhibition of protein S-nitrosylation by epigallocatechin gallates in lipopolysaccharide-stimulated microglial cells
Source: J Neuroinflammation. 2014 Jan 28;11:17. doi: 10.1186/1742-2094-11-17 (PMC3922161; doi:10.1186/1742-2094-11-17)

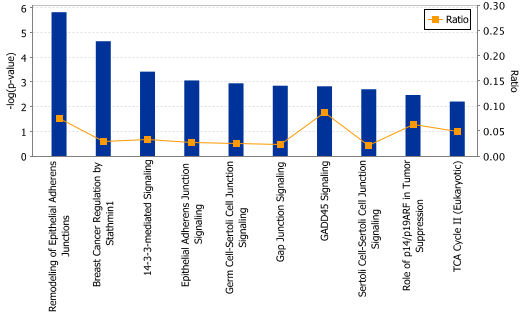

Supplement: Additional file 2: Figure S1 — IPA analysis of protein S-nitrosylation in ex vivo SNOC-treated BV-2 cells. A total of 67 SNO-proteins were identified from SNOC-treated BV-2 cells and the top 10 canonical pathways involved by these proteins were predicted by IPA analysis. [file 1742-2094-11-17-S2.tiff]

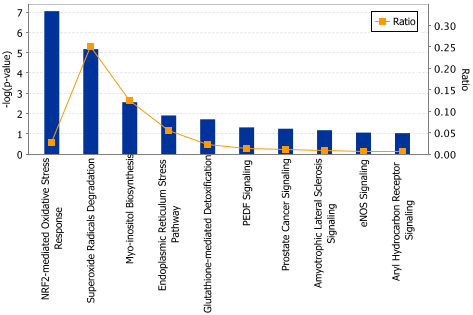

Supplement: Additional file 4: Figure S2 — IPA analysis of protein S-nitrosylation in LPS-stimulated BV-2 cells. In total, 13 SNO-proteins were identified from LPS-stimulated BV-2 microglial cells, and the top 10 canonical pathways participated by these proteins were predicted by IPA. [file 1742-2094-11-17-S4.tiff]
